# Exposure to perfluoroalkyl substances in early pregnancy and risk of sporadic first trimester miscarriage

**DOI:** 10.1038/s41598-021-82748-6

**Published:** 2021-02-11

**Authors:** Sverre Wikström, Ghada Hussein, Annika Lingroth Karlsson, Christian H. Lindh, Carl-Gustaf Bornehag

**Affiliations:** 1 School of Medical Sciences, Örebro University, Södra Grev Rosengatan 30, 703 62 Örebro, Sweden; 2 Department of Obstetrics and Gynecology CLINTEC, Karolinska institute, Stockholm, Sweden; 3 Stockholm IVF-EUGIN Clinic, Stockholm, Sweden; 4 Division of Occupational and Environmental Medicine, Lund University, Lund, Sweden; 5 Department of Health Sciences, Karlstad University, Karlstad, Sweden; 6 Department of Preventive Medicine, Icahn School of Medicine at Mount Sinai, New York City, USA

**Keywords:** Environmental chemistry, Medical research, Epidemiology

## Abstract

Many first trimester sporadic miscarriages are unexplained and the role of environmental exposures is unknown. The present aim was to study if levels of Perfluoroalkyl substances (PFASs) in early pregnancy are associated with unexplained, sporadic first trimester miscarriage. The study was performed within the Swedish SELMA pregnancy cohort. Seventy-eight women with non-recurrent first trimester miscarriage were included and 1449 women were available as live birth controls. Eight PFASs were measured in first trimester serum. A doubling of perfluorooctanoic acid (PFOA) exposure, corresponding to an inter-quartile increase, was associated with an odds ratio (95%CI) for miscarriage of 1.48 (1.09–2.01) when adjusting for parity, age and smoking. Analyses per quartiles of PFOA exposure indicated a monotonic dose response association with miscarriage. A similar, but not significant, pattern was observed for perfluorononanoic acid (PFNA). For other PFAS, there were no associations with miscarriage. We have previously shown associations between early pregnancy PFAS exposures and preeclampsia, as well as lower birth weight. Now we report an association between PFOA and miscarriage within the same cohort, which may suggest shared but unknown mechanisms. The study can only represent a period of early placentation and clinical pregnancy loss during the second half of the first trimester.

## Introduction

Early gestation is established after a complex biochemical dialogue between the endometrium and blastocyst. Any defect in this interaction and milieu may result in adverse pregnancy outcomes including miscarriage. Sporadic first trimester miscarriage (up to gestational weeks 12 + 6) is a distressing and common complication that ends about 15–20% of pregnancies^1,2^. Previous studies suggest a multifactorial background where genetic factors, advanced maternal age, endocrine or immunological dysregulation, lifestyle including smoking^3^ and sperm DNA integrity may play a role^1^. Still, a large number of first trimester sporadic miscarriages are unexplained^1^ and studies on the importance of environmental chemical exposures for fetal loss within this group is warranted^4^.

The manmade perfluoroalkyl substances (PFASs) make surfaces repellent to fat, water and dirt, which is useful in a wide variety of consumer products. Examples where PFASs are used include fabrics and all weather clothing, grease proof paper, ski wax, non-stick materials and fire-fighting foam^5^. PFASs are widespread and biomonitoring data show that almost everyone is exposed^6^. PFAS are also very persistent in the environment and have long half-lives in humans^7^. Therefore, human levels of PFASs can be expected to decrease only slowly after voluntary and regulatory efforts.

In human, a continuously increasing number of publications reports associations between higher serum levels of several PFASs during pregnancy, especially perfluorooctane sulfonate (PFOS) together with perfluorooctanoic acid (PFOA), and preeclampsia^8–10^ as well as reduced fetal growth^11–14^. Causation for such associations has however been questioned^13^. These unfavorable pregnancy outcomes however share in common their relation to placental dysfunction, and there is experimental support for effects from PFAS on placentation^15,16^. This makes it important to study also associations between PFAS exposure and miscarriage, since abnormal placentation is important in the aetiology also of miscarriage. Despite this, human investigations of PFAS exposure as risk factor of miscarriage are limited^8,10,17–20^ and findings are highly divergent.

The aim of this study was hence to investigate whether maternal exposure to eight PFASs in early pregnancy is associated with unexplained, sporadic first trimester miscarriage of the same pregnancy.

## Results

**Figure 1:**
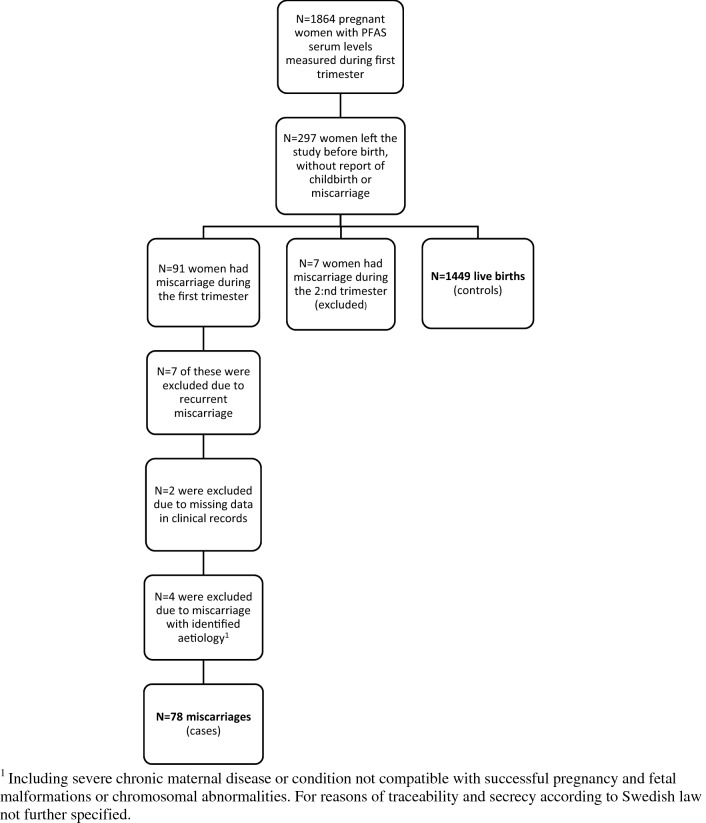
Description of inclusion and exclusion procedure.

In total 2,582 pregnancies were included in the SELMA study. After inclusion of only one pregnancy per participant if a woman was recruited at multiple times (e.g. after miscarriage); restriction to women with blood samples feasible for PFAS and cotinine analysis sampled during the first trimester; and exclusion of cases with any inconsistencies in study data as well as all twin pregnancies, a number of 1864 pregnant women remained eligible for inclusion in the present investigation. We identified 91 women with early pregnancy (i.e., first trimester) analysis of PFASs suffering a first trimester miscarriage. Of these, 78 women were included as cases according to our criteria. For 1449 of the 1864 women there were data on child birth, making them available as live birth controls. See Fig. 1 for a graphical description of the inclusion and exclusion procedure and Table 1 for basal data on the study population.

**Table 1:** Description of the study population of miscarriage cases (N = 78) and singleton live birth (N = 1449).

Age (years)		Miscarriage n (%)	Live birth n (%)	p-value
		31 (29–35)	31 (28–34)	0.157
Parity	Nulliparous	40 (51.3)	684 (47.2)	0.488
	I-para	22 (28.2)	517 (35.7)	0.178
	II-para +	16 (20.5)	248 (17.1)	0.439
Tobacco smoke-exposure	Active smoking	6 (7.7)	105 (7.2)	0.480
	Passive smoking	6 (7.7)	86 (5.9)	0.340
	Non smoking	66 (84.6)	1258 (86.8)	0.290

Numbers are presented as median (IQR) for age and N (%) for parity and smoke exposure.

P-values for difference between miscarriage cases and live birth pregnancies.

Blood (serum) for PFAS analyses, was sampled at median (IQR) 10 (9–10) gestational weeks in both miscarriage cases and controls (p = 0.50). The time of fetal death was however dated by ultrasonography to median (IQR) 8 (7–11) weeks of pregnancy. The explanation for this is that miscarriage can be seen as a process where fetal death may proceed fetal expulsion and clinical signs of miscarriage, in other words meaning that 36 women actually had their miscarriage already before they were recruited to the SELMA study, however, without knowing it. The distributions of the serum concentrations of PFAS are presented in Table 2. Due to > 50% of samples with concentrations below LOD, we excluded PFDoDA from the analyses.

**Table 2:** Distribution of first trimester serum concentrations (ng/mL) of PFASs.

Compound	Miscarriage, n = 78				Live births, n = 1449			
	Geometric mean (95% CI)	Median (IQR)	95:th perc	> LOD %	Geometric Mean (95% CI)	Median (IQR)	95:th perc	> LOD %
PFOS	5.68 (5.03–6.42)	6.09 (3.99–8.77)	13.12	100	5.40 (5.26–5.55)	5.45 (4.00–7.68)	12.57	100
PFOA	1.89 (1.68–2.12)	2.00 (1.44–2.76)	4.08	100	1.63 (1.58–1.68)	1.64 (1.13–2.32)	4.09	100
PFHxS	1.28(1.13–1.46)	1.21 (0.83–2.01)	3.31	100	1.31 (1.27–1.35)	1.23 (0.86–1.96)	3.70	100
PFNA	0.54 (0.53–0.56)	0.59 (0.43–0.91)	1.78	100	0.60(0.53–0.68)	0.53 (0.39–0.74)	1.33	100
PFDA	0.26 (0.23–0.30)	0.27 (0.19–0.38)	0.72	100	0.26 (0.26–0.27)	0.26 (0.19–0.34)	0.61	100
PFUnDA	0.21 (0.18–0.25)	0.23 (0.16–0.32)	0.55	100	0.21 (0.21–0.22)	0.23 (0.15–0.32)	0.54	99.7
PFHpA	0.02 (0.02–0.02)	0.02 (0.01–0.04)	0.12	76.9	0.02 (0.02–0.02)	0.02 (0.01–0.04)	0.10	74.7

LOD, limit of detection.

**Figure 2:**
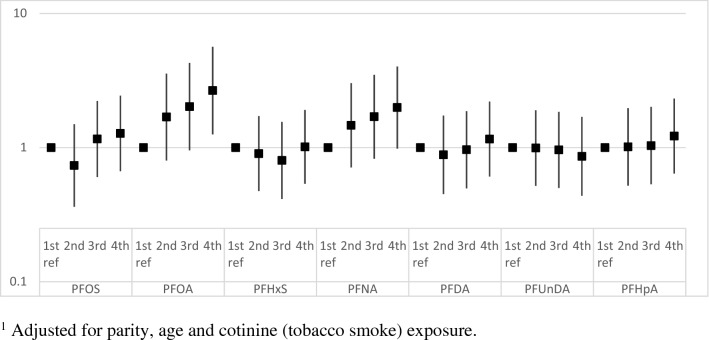
Adjusted associations^1^ (Odds Ratios with 95% Confidence Intervals) between seven PFASs and miscarriage, presented by quartiles of exposure with respectively first quartile exposure as reference.

Higher serum level of PFOA was associated with increased risk for miscarriage in crude analysis and also when adjusting for age of the mother, parity and tobacco smoke exposure during pregnancy. A one unit increase in log base-2 PFOA exposure, i.e. doubling of exposure, was associated with an OR(95%CI) for miscarriage of 1.48 (1.09–2.01). Crude and adjusted associations between PFAS concentrations and miscarriage are presented in Table 3**.** When analyzed per quartiles of exposure and adjusted for potential confounders (Fig. 2), there was a significant increase in risk for miscarriage within the highest quartile of PFOA with OR(95%CI) = 2.66 (1.26–5.65), as compared with first quartile exposure. In the third quartile OR (95% CI) was 2.02 (0.95–4.29) and in the second quartile 1.69 (0.80–3.56). A similar, non-significant, pattern was observed for PFNA (Fig. 2) with OR(95%CI) = 1.99 (0.98–4.02) in the upper quartile as compared with first (p = 0.056). For other PFAS compounds there were no associations with miscarriage.

**Table 3:** Crude and adjusted associations between early pregnancy serum levels of PFASs and miscarriage.

Compound	Crude OR (95% CI)	Adjusted^a^ OR (95%CI)
PFOS	1.13 (0.84–1.53)	1.13 (0.82–1.52)
PFOA	1.38 (1.04–1.83)*	1.48 (1.09–2.01)*
PFHxS	0.96 (0.73–1.26)	0.96 (0.73–1.26)
PFNA	1.25 (0.94–1.66)	1.25 (0.93–1.68)
PFDA	1.14 (0.84–1.55)	1.10 (0.81–1.53)
PFUnDA	1.00 (0.78–1.28)	0.95 (0.74–1.22)
PFHpA	1.09 (0.93–1.27)	1.09 (0.93–1.28)

Odds ratios; 95%CI calculated per one unit increase in log base-2 PFAS serum levels (i.e. doubling of exposure).

^a^Adjusted for parity, age and cotinine (tobacco smoke) exposure.

*Significant association at p < 0.05 level.

When stratifying for parity, a one unit increase in log base-2 PFOA was associated with an OR (95%CI) of 1.22 (0.79–1.89) in nulliparous women (n = 40) and 1.60 (1.02–2.52) in parous (n = 38), adjusted for age and smoke exposure as above. For other PFAS compounds, we found no significant association with miscarriage in analyses stratified by parity (Supplementary Tables 1 and 2). If parity was instead omitted from the predictors in multiple logistic regression (hence only adjusting for age and cotinine, *n* = 78 miscarriage cases), one unit increase in PFOA was associated with an odds ratio (95%CI) for miscarriage of 1.45 (1.09–1.92). In the sensitivity analysis excluding miscarriages in women with hypothyroidism, IBD or diabetes (n = 4), risk estimates were unchanged with OR(95%CI) = 1.48 (1.08–2.01) for one unit increase in log base-2 PFOA.

## Discussion

In this pregnancy cohort, we observed an association between PFOA serum levels in early pregnancy and increased risk for sporadic miscarriage during the second half of the first trimester, this adjusted for parity, age, and tobacco smoke. A doubling of the PFOA exposure, corresponding to an increase from 25:th to 75:th percentile of exposure, was associated with around 50% increased risk for miscarriage in adjusted analyses, with indications of a monotonic dose response association.

The positive association between early pregnancy PFOA exposure and miscarriage during the first trimester is a novel finding. Recently, a nested case–control study within the Danish National Birth Cohort, DNBC (sampled during years 1996 through 2002), reported an association between maternal PFOA and second trimester miscarriage^20^. The association was strongest among parous women, when investigating in total n = 220 cases of miscarriage. Our findings from the second half of the first trimester were very similar to the second trimester findings of the Danish report: From the DNBC, Liew et al. reported that the overall OR(95%CI) for miscarriage with a doubling of PFOA exposure was 1.4 (1.0–1.9), to be compared with OR(95%CI) = 1.48 (1.09–2.01) in the present cohort. Furthermore, the Danish study reported an odds ratio for miscarriage in the highest quartile of PFOA exposure of 2.2(95% CI: 1.2- 3.9), as compared with reference^20^. The corresponding OR in our adjusted analyses of the fourth quartile of PFOA was 2.66 (1.26–5.65), and signs of monotonic dose response was observed in both cohorts.

Other studies on PFAS exposure in relation to miscarriage^8,10,17–19^ identified no such associations for PFOA. Regarding PFAS compounds other than PFOA, the DNBC case–control study reported an increase in odds for miscarriage in the highest quartile of perfluoroheptane sulfonate (PFHpS)^20^. This substance was not analysed in our study. Darrow et al.^19^ previously demonstrated a modest association between PFOS and miscarriage in a sub group analysis within the *C8 Health Project.* This was however not replicated in our study. Earlier findings on PFNA are divergent. Jensen et al. showed a marked increase in risk for miscarriage with an odds ratio of 16.17(95%CI: 6.88–38.03) for women in the highest tertile of exposure as compared with lowest tertile^17^. Also PFDA was there associated with miscarriage^17^. Louis et al., on the contrary, reported significant pregnancy loss risk reduction with Hazard Ratio (95%CI) 0.57(0.34–0.94) at PFNA exposures in the third versus first tertile^18^. Our lack of statistical significance for the positive associations also between  miscarriage and PFNA (p = 0.136 and p = 0.056 in the respectively analyses, Table 3 and Fig. 2) could be a matter of statistical power. However, we find that it may otherwise be explained by strong inter-correlation between PFOA and PFNA serum levels as already reported in from the same cohort^9^. PFAS compounds in general show inter- correlated serum concentrations due to common sources of exposure. Therefore, mixture-based approaches are requested in further studies.

The reasons for the diverging results in associations from the limited number of studies of this emerging research question are not clear. Even though PFAS exposure levels differ between the studies, PFAS serum concentrations provide no obvious explanation of the discrepancies in findings. The *C8 Health Project* study population is all-over highly exposed for PFOA and likewise, the *LIFE* Study (USA) reports more than twice the PFOA levels of ours^18^ and also the larger Danish National Birth Cohort study^20^ represents (historically) higher exposures to PFOA than in the present cohort. This may blunt possible threshold effects present already at lower exposure levels. Differences in exposure levels are, however, unlikely to be the full explanation for different results. This especially since exposure levels of the Danish Odense cohort, with no association between PFOA and miscarriage^17^, are in the same low exposure level range as ours. Still, studies in highly exposed populations could suffer from selection bias if there is an association between PFOA exposure and reduced fertility, which may possibly reduce the number of conceived pregnancies among women especially vulnerable to reproductive effects from PFAS exposure^21^. The support for a causal relation between PFOA and female fecundability is however very weak^22^.

The identification of miscarriage cases and the pregnancy length at which they occur constitute clear differences between different studies. In the present study all miscarriages were verified by ultrasound and were dated to the second half of the first trimester. The U.S. based *C8 Health Project* studies based their analyses on self- reported pregnancies (according to telephone interview)^19^, and in the sample reported by Darrow et al., where an association between PFOS (but not PFOA) and miscarriage was identified, 17% of the 1438 pregnancies ended in spontaneous loss before 20 gestational weeks^19^. The *LIFE study* followed 344 women from preconception recruiting and investigated the association between preconception PFAS serum levels and pregnancy loss, as determined by conversion of pregnancy tests (n = 98, constituting 28% of the women in the study group), rather than clinical diagnosis of miscarriage^18^. In the Danish Odense cohort, women were recruited at 8–16 weeks of pregnancy, serum measures of PFASs were collected before week 12, and miscarriage (n = 56) as verified in hospital occurred at maximum 22 weeks of gestation^17^. Liew et al. in the DNBC study^20^ utilized samples collected in the first trimester and investigated associations with second trimester miscarriage, based on data obtained from a Hospital Discharge Register.

Of great importance and different from previous studies, the focus of our study was on unexplained sporadic first trimester clinical miscarriage and hence we excluded all later cases, or when a specific aetiology was identified in clinical care. Instead of statistical adjustment for previous miscarriage we considered recurrent pregnancy loss (defined as three or more sequentially miscarriages, in line with the guidelines of Royal College of Obstetricians and Gynaecologists) an exclusion criterion. All together, the strict inclusion/exclusion criteria of the present study may explain some difference from other cohorts reported. Another explanation is the narrow span in gestational time of the studied miscarriages (as result of the study design): The aetiology may differ considerably^1^ between the very common earliest pregnancy losses possible to identify when pre-conception recruitment is utilized, our clinical cases dated to 6–12 weeks, and finally miscarriages occurring during second trimester. Well defined causes of miscarriage, not associated with chemical exposure but more often present during specific parts of pregnancy (e.g. embryonic chromosomal aetiology during early gestation), may draw associations towards the null if inclusion criteria are wide. For recurrent miscarriage, risk factors are also much more well-established^1^, and in itself constituting an important statistical risk factor for achieving future miscarriages. The present study may only represent clinical miscarriages during a period of early placentation.

Several potential confounders must be considered. We adjusted the analyses for age and tobacco smoke exposure, established risk factors for miscarriage, also associated with PFAS serum concentrations^6,23^. The higher percentage of nulliparous women among the women with miscarriage as compared to the live birth group (51% vs 46%) was not statistically significant. Despite this, and despite the similar odds ratios achieved without adjustment for parity, we regard parity the most important potential confounder of the present study. When we stratified women into parous and nulliparous, the association between PFOA exposure and miscarriage remained significant in the parous group, despite the small sample size (n = 38). This may indicate a risk of residual confounding from factors related to reproductive history. Inter-pregnancy interval, for which we could not control, is associated with re-accumulation of PFAS after delivery^24^. But since the association between inter-pregnancy interval and miscarriage is controversial^25^, we do not consider this as an important source of reverse causation bias. The association in parous but not nulliparous women could as well indicate a higher rate of women with other, un-identified, causes of both subfertility and pregnancy loss in the nulliparous group. This group consists of both first-time pregnant women and women with previous unsuccessful pregnancies and the present study sample is likely underpowered for analyses of such a heterogeneous group. We regard this a potential of bias towards the null for associations between PFAS and miscarriage in the full cohort.

We did not adjust analyses for pregnancy related changes in glomerular filtration rate or hemodynamics. The present serum samples were however drawn at median 10 weeks of gestation and we therefore find a very minor risk of confounding^26^. We had no data available on occupation, and especially night shift work may be a risk factor for miscarriage^27^, but we have found no previous evidence of associations between such work in general and PFAS levels (outside very specific industries with occupational exposures)^28^.

Our low (6%) miscarriage rate in comparison with previous studies on PFASs and miscarriage, is likely explained by the restriction to first trimester miscarriage, but most important also by the study recruitment procedure implemented in clinical antenatal care routine. Due to the study design we most likely missed out a considerable number of early miscarriages, which are also the most common. Although the women provided blood samples in median at 10 weeks of pregnancy, fetal death had overall actually occurred in weeks 6–12. Hence fetal death somewhat proceeded blood sampling in 36 cases, although without clinical signs of miscarriage yet (i.e., signs of bleeding and fetal expulsion). We have no reason to believe that these women had different aetiology of their miscarriage than women presenting with miscarriage with slightly shorter delay until clinical symptoms, nor that this short period of carrying a dead fetus would affect the PFAS exposure measurements, all sampled in the first trimester. PFAS half-lives in humans are in general several years^7^. Although we acknowledge that this design implies a potential source of selection bias, we consider it unlikely to influence the validity of our results. We find no reason to suspect confounding by gestational age at blood sampling. Timing of samples was the same among women with miscarriage and live birth, and in the full cohort altogether, there was no association between gestational age at sampling and PFOA level (r = -0.012; p = 0.912). A final limitation to the present study, shared with many others, is the lack of control for the male factor in fetal loss, which may also be studied in future investigations on environmental exposures.

The possible mechanisms behind an association between PFOA exposure and miscarriage are unknown. The building evidence of associations between PFAS exposures and adverse human reproduction outcomes including miscarriage, preeclampsia and impaired fetal growth suggest shared mechanisms. Although manifest at different stage of pregnancy, these endpoints share in common the strong association with abnormal placentation, deficient trophoblast invasion being an important part of the mechanisms. Unifying the three pregnancy outcomes is also placental oxidative stress. Experimental findings are therefore interesting, where PFAS compounds may disrupt placental endocrine function^15,16^, possibly via induction of apoptosis in human placental syncytiotrophoblasts, with effects on placental development^16^. Also, that PFASs can increase reactive oxygen species (ROS) generation in vitro. Cells exposed to PFOA were found to have a significant lower antioxidant capacity^29^ and PFOS induced ROS generation in human first trimester trophoblast^30^.

## Conclusions

In the SELMA-study we have previously shown associations between early pregnancy PFAS exposures, including PFOS, PFOA and PFNA, and preeclampsia as well as lower birth weight^9,14^. Within the same cohort we now add the novel finding of a positive association between first trimester PFOA exposure and increased risk for sporadic miscarriage during the second half of the first trimester.

## Methods

### Study group

The study was embedded in the Swedish Environmental Longitudinal, Mother and child, Asthma and allergy (SELMA) study. SELMA is a prospective pregnancy cohort, designed to investigate children´s health and development as well as reproductive outcomes in relation to early pregnancy chemical exposures (including eight PFAS compounds)^6,31^. Pregnant women were recruited between September 2007 and March 2010, adjacent to their enrolment in antenatal care during early pregnancy. The study was performed in accordance with the Declaration of Helsinki and the Regional Ethical Review Board in Uppsala, Sweden approved the study protocol. Written informed consent was obtained from each participating woman.

Eligible for the present investigation were women with first trimester PFAS serum analysis and data on childbirth or miscarriage confirmed by clinical records. All twin pregnancies (N = 16) were excluded from analysis.

For all originally recruited women who left the study before registered childbirth, including early study drop-out for any reason, midwives in antenatal care returned a form where miscarriage was one among the specified causes for leaving. This was considered to reduce the probability of a having a high proportion of miscarriage cases in the group of women leaving the study for unknown reasons. Reported cases of miscarriage were validated as intrauterine miscarriages according to clinical patient records, in all cases including ultrasound investigations. Gestational age by the time of miscarriage was based on the ultrasound dating, or if not possible so, on last menstrual period. Since time of fetal death was in some cases dated backwards from the clinical presentation, the gestational age at miscarriage could during such circumstances be dated to before study enrolment. Inclusion was restricted to miscarriages during the first trimester (i.e., before 12 weeks + 6 days of pregnancy).

In line with the aim to study sporadic first trimester miscarriage of unknown aetiology, we excluded women previously having more than three consecutive pregnancy losses. This is the present definition of recurrent pregnancy loss according to the Royal College of Obstetricians and Gynaecologists^32^, also constituting a threshold for the number of miscarriages where the different international guidelines agree that investigation and counselling is (by the latest) warranted.

Without knowledge of individual PFAS levels we reviewed clinical records to identify miscarriages obviously explained by other defined maternal conditions (severe chronic disease or uterine condition not compatible with successful pregnancy) or fetal (identified malformation or chromosomal abnormality) which were excluded from analysis. We also decided a priori to exclude women with chronic kidney disease because of the increased risk for miscarriage and concomitant but complex effect on serum PFAS levels^33,34^.

### Chemical analysis of serum

Blood samples were collected at enrolment to the study, for all women in the present study group during the first trimester and at median 10 weeks of gestation The current data were collected within the prospective pregnancy cohort study, where all samples were analyzed in random order with no knowledge about any outcome measure. Eight PFASs were measured in serum: PFOS, PFOA, perfluorohexane sulfonate (PFHxS), perfluorononanoic acid (PFNA), perfluorodecanoic acid (PFDA), perfluoroundecanoic acid (PFUnDA), perfluoroheptanoic acid (PFHpA) and perfluorododecanoic acid (PFDoDA). The serum samples were analyzed using a liquid chromatography—triple quadrupole linear ion trap mass spectrometer (LC/MS/MS; QTRAP 5500, AB Sciex, Foster City, CA, USA) at the Department of Occupational and Environmental Medicine in Lund Sweden, according to modified method^35^. In brief, aliquots of 100 μl serum were added with labelled internal standards for all measured compounds. Proteins were precipitated by acetonitrile and vigorously shaking for 30 min prior to analysis. In all sample batches, chemical blanks and quality control (QC) samples, were included. The analyses of PFOS and PFOA are part of a quality control program between analytical laboratories coordinated by University of Erlangen-Nuremberg, Germany. In addition, the laboratory participates in the HBM4EU QA/QC program and is qualified as HBM4EU laboratory for the analysis of all the PFAS included in the present study. Compounds with < 50% of samples above limit of detection (LOD) were excluded from further analyses where values below LOD were set to half the LOD.

As a measurement of tobacco or nicotine exposure, we analyzed cotinine in serum using LC/MS/MS as previously described^35^. Subjects were categorized as non-smokers if their serum cotinine levels were below the limit of Detection (LOD) of 0.2 ng/mL, passive smokers at levels 0.2-15 ng/mL and active smokers at cotinine levels above 15 ng/mL^36^.

### Statistical analyses

Serum PFAS and cotinine concentrations were transformed with the base-2 logarithm for approximation of normal distribution. Non-parametric tests were applied to compare background data between miscarriage cases and live birth controls. Multiple logistic regression models were applied, compound by compound, for analyses of PFASs as predictors of miscarriage, with calculation of Odds Ratios (OR) for miscarriage. This was performed per one-unit increase in log base-2 PFAS concentrations (i.e. doubling of exposure), and also per quartiles of exposure with the first quartile as reference.

As co-variates in the adjusted models of multiple regression we included age, cotinine levels (log base-2 cotinine) reflecting tobacco exposure and parity (nulliparous, I-para, II-para + , as reported at enrolment), since we a priori identified them important as potential confounders in the association between PFAS exposure and miscarriage^1,2,6,23^. All study data collection directly from participants (e.g. questionnaires) in SELMA ended when a woman left the study, including when due to miscarriage. Therefore, data regarding minor risk factors for miscarriage, e.g. occupation, education level or pre pregnancy BMI^27^were not available the all miscarriage cases and hence omitted from regression analysis.

Parity is a strong determinant of PFAS serum levels^6^ and also a function of previous successful pregnancies. Analyses were therefore in a second analysis also stratified into nulliparous and parous women. Complementary sensitivity analyses were performed, excluding women with hypothyroidism, Inflammatory Bowel Disease or diabetes (according to clinical records) among women with miscarriage (similar standardized clinical data were not available for women with live births). For the statistical analyses IBM SPSS Statistics for Windows, Version 25 (NY,IBM Corp, USA) was used.

## Supplementary Information

Supplementary Information.

## Data Availability

Data on chemical exposures (PFAS and cotinine) can be made available to researchers upon request (subject to a review of secrecy). Requests for data should be made to the Head of Department of Health Sciences, Karlstad University. However, according to the Ethical Review Board decision and obtained personal consent, clinical data cannot be made freely available. This since as they are subject to secrecy in accordance with the Swedish Public Access to Information and Secrecy Act [OSL 2009:400]. Unique combinations of such data will make a study participant (i.e. patient) identifiable, and consequently no clinical data will be shared.
