# Supplementary material for: Exposure to perfluoroalkyl substances in early pregnancy and risk of sporadic first trimester miscarriage
Source: Sci Rep. 2021 Feb 11;11:3568. doi: 10.1038/s41598-021-82748-6 (PMC7878909; doi:10.1038/s41598-021-82748-6)
Supplement: Supplementary file 1 — Supplementary Information. [file 41598_2021_82748_MOESM1_ESM.pdf]

Exposure to perfluoroalkyl substances in early pregnancy and risk of sporadic first trimester miscarriage

Sverre Wikström, Ghada Hussein, Annika Lingroth Karlsson, Christian H. Lindh, Carl-Gustaf Bornehag

Supplementary Table 1. *Background data on age, tobacco smoke exposure and first trimester PFAS serum concentrations (ng/mL) in the full sample of women with miscarriage or live births, stratified by parity.*

		Nulliparous N=803	Parous N=724	p-value
Age (years)		29(26-32)	33(30-35)	<0.001
Tobacco smoke-exposure	Active/passive smoking	93(13%)	110(14%)	0.651
	Non smoking	631(87%)	693(86%)	
PFOS		6.31(4.65-8.74)	4.81(3.59-6.74)	<0.001
PFOA		2.17(1.60-2.84)	1.33(0.94-1.76)	<0.001
PFHxS		1.36(0.99-2.20)	1.08(0.78-1.77)	<0.001
PFNA		0.61(0.45-0.85)	0.47(0.35-0.65)	<0.001
PFDA		0.28(0.21-0.38)	0.24(0.18-0.31)	<0.001
PFUnDA		0.24(0.16-0.35)	0.22(0.14-0.30)	0.001
PFHpA		0.02(0.01-0.04)	0.02(0.01-0.03)	<0.001

Supplementary Table 2. *Crude and adjusted associations between early pregnancy serum levels of PFASs and miscarriage, stratified by parity. Odds Ratios(95%CI) calculated per one unit increase in log base-2 PFAS serum levels (i.e doubling of exposure).*

	Nulliparous n=40	Parous n=38
Compound	Adjusted[1] OR (95%CI)	Adjusted[1] OR (95%CI)
PFOS	1.17 (0.76-1.80)	1.0 (0.64–1.55)
PFOA	1.22 (0.79-1.89)	1.60 (1.02-2.52)*
PFHxS	1.10 (0.74-1.61)	0.78 (0.52-1.17)
PFNA	1.21 (0.81-1.80)	1.21 (0.77-1.89)
PFDA	1.11 (0.72-1.71)	1.03 (0.65-1.66)
PFUnDA	0.99 (0.69-1.42)	0.88 (0.62-1.21)
PFHpA	0.98 (0.79-1.22)	1.21 (0.96.1.53)

1) Adjusted for age and cotinine (tobacco smoke) exposure.

*) Significant association at p <0.05 level
